# The Evolving Reconstruction Toolbox of the Plastic Surgeon

**DOI:** 10.1055/a-2716-4238

**Published:** 2025-11-20

**Authors:** Hyunsuk Peter Suh

**Affiliations:** 1 Department of Plastic and Reconstructive Surgery, Asan Medical Center, University of Ulsan, College of Medicine, Seoul, Republic of Korea

**Figure:**
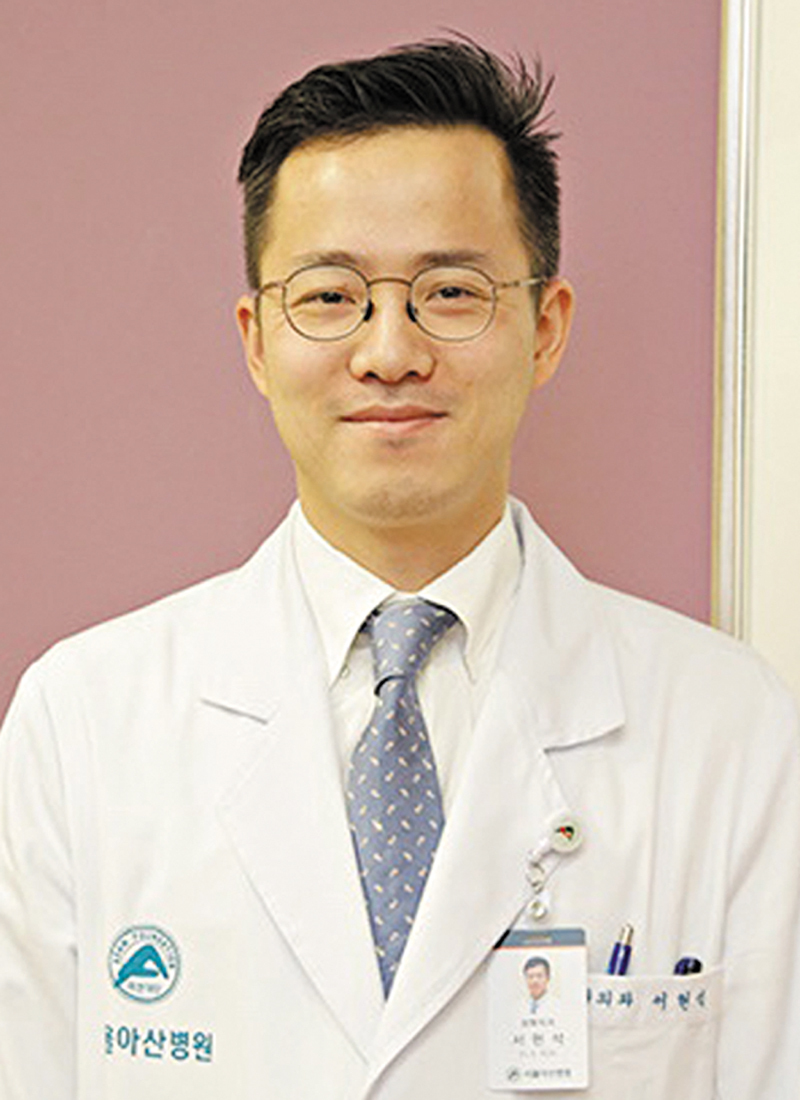
Hyunsuk Peter Suh: Editor-in-Chief

For decades, reconstructive plastic surgery has been guided by the paradigm of the “reconstruction ladder.” This stepwise algorithm emphasized beginning with the simplest modality—such as primary closure or skin grafting—and ascending gradually to more complex techniques, including local, regional, and free tissue transfer. While this framework provided a structured pathway, it often encouraged the use of less effective methods merely because they occupied a lower rung on the ladder.

**Fig. 1:**
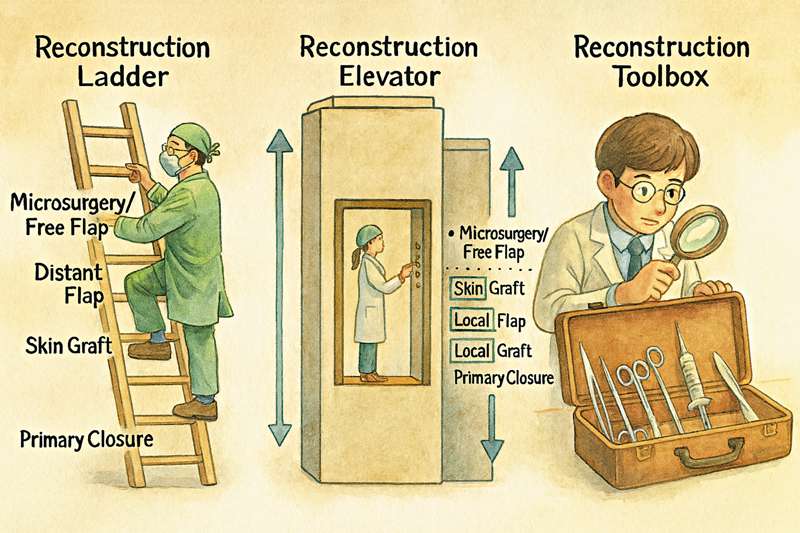
From algorithms to action: the evolution of reconstruction and the surgeon's toolbox.

The subsequent concept of the “reconstruction elevator” challenged this rigidity by allowing the surgeon to select the most appropriate reconstructive option directly, regardless of its perceived complexity ( Fig. 1 ). This model promoted individualized decision-making, weighing functional outcomes, donor site morbidity, and complication risks. Nevertheless, the elevator concept presupposes that every surgeon is equally equipped to perform the full spectrum of procedures. In practice, this is rarely the case. A newly trained surgeon may not yet possess microsurgical expertise, and seasoned practitioners often develop preferences for particular methods.

In reality, each reconstructive surgeon operates with a unique “toolbox,” shaped by training, experience, institutional resources, and personal refinement. A well-equipped toolbox should encompass local flaps, regional and muscle flaps, perforator and free flaps, tissue expansion, and mastery of skin grafting. Importantly, the toolbox must also include principles of wound management. Negative pressure wound therapy, delayed closure, secondary intention healing, and a sound understanding of wound biology often constitute the most effective interventions. Reconstructive surgery, at its core, begins not with flap transfer but with the management of an open wound.

The early years of independent practice are characterized by the active process of filling the toolbox. Surgeons must refine fundamental techniques, gain confidence with free tissue transfer, and learn to handle complications with consistency. As practice matures, the emphasis transitions from acquisition to refinement. While many surgeons achieve reliable outcomes within 5 to 10 years, true mastery lies beyond the absence of major complications. It involves elevating thinner, safer flaps, achieving survival rates approaching 100%, and minimizing morbidity to the greatest possible extent.

Over time, the evolution of the toolbox may paradoxically lead to its simplification. Surgeons may rely on fewer techniques, but those retained are honed to extraordinary precision and reliability. In this sense, the career of a reconstructive microsurgeon is not simply an ascent up a ladder nor a ride in an elevator, but rather a lifelong journey of constructing, refining, and perfecting one's toolbox.

Thus, each surgeon must continually ask: *What tools are in my reconstruction toolbox, and which of them do I wield with the greatest mastery?*

